# Engineering RNA Virus Interference via the CRISPR/Cas13 Machinery in *Arabidopsis*

**DOI:** 10.3390/v10120732

**Published:** 2018-12-19

**Authors:** Rashid Aman, Ahmed Mahas, Haroon Butt, Zahir Ali, Fatimah Aljedaani, Magdy Mahfouz

**Affiliations:** Laboratory for Genome Engineering, Division of Biological Sciences, 4700 King Abdullah University of Science and Technology, Thuwal 23955-6900, Saudi Arabia; rashid.aman@kaust.edu.sa (R.A.); ahmed.mahas@kaust.edu.sa (A.M.); Haroon.butt.1@kaust.edu.sa (H.B.); zahir.ali@kaust.edu.sa (Z.A.); fatimah.aljedaani@kaust.edu.sa (F.A.)

**Keywords:** CRISPR/Cas systems, CRISPR/Cas13a, virus interference, RNA interference, molecular immunity, RNA knockdown, transcriptome regulation

## Abstract

Clustered regularly interspaced short palindromic repeats (CRISPR) and CRISPR-associated (Cas) systems are key immune mechanisms helping prokaryotic species fend off RNA and DNA viruses. CRISPR/Cas9 has broad applications in basic research and biotechnology and has been widely used across eukaryotic species for genome engineering and functional analysis of genes. The recently developed CRISPR/Cas13 systems target RNA rather than DNA and thus offer new potential for transcriptome engineering and combatting RNA viruses. Here, we used CRISPR/LshCas13a to stably engineer *Arabidopsis thaliana* for interference against the RNA genome of Turnip mosaic virus (TuMV). Our data demonstrate that CRISPR RNAs (crRNAs) guiding Cas13a to the sequences encoding helper component proteinase silencing suppressor (HC-Pro) or GFP target 2 (GFP-T2) provide better interference compared to crRNAs targeting other regions of the TuMV RNA genome. This work demonstrates the exciting potential of CRISPR/Cas13 to be used as an antiviral strategy to obstruct RNA viruses, and encourages the search for more robust and effective Cas13 variants or CRISPR systems that can target RNA.

## 1. Introduction

Plant viruses invade their host plants to replicate and propagate, severely affecting plant growth and yield, causing significant losses in crop quality and quantity, and thus seriously threatening food security around the world [1,2,3,4]. Combatting such devastating losses requires efforts to develop novel technologies to control plant viruses. One of the best solutions to protect plants is to improve their resistance against viruses via plant breeding or targeted trait engineering. Different strategies to engineer virus resistance have been developed over the years, including transgenic expression of viral and non-viral factors [5,6,7,8,9]. Although these technologies are promising, many hurdles have complicated their utility in agriculture [10,11].

Genome engineering is a powerful tool that can be used to improve plant resistance against viruses. Clustered regularly interspaced short palindromic repeats (CRISPR) and CRISPR-associated (Cas) proteins are adaptive immunity systems that protect bacteria and archaea from invasions of phages and other RNA and/or DNA genetic elements [12]. Recently, unique and flexible genome engineering platforms based on CRISPR/Cas systems have been developed [13,14]. Among these, Class II CRISPR/Cas systems are the most widely developed and used systems for RNA and DNA targeting and regulation, owing to their dependence on two simple components, a Cas effector protein that mediates cleavage of the target RNA or DNA, and a guide RNA (gRNA) that guides the Cas protein to the target nucleic acid via Watson–Crick base pairing [15]. The great success of CRISPR/Cas technology in plant breeding to develop plants with desirable traits has inspired the application of this technology to engineer plants with durable resistance to many viruses [16]. The CRISPR/Cas9 technology has already been proven to efficiently confer resistance to host plants against several major plant DNA viruses [17,18,19,20]. However, the majority of plant pathogenic viruses possess RNA genomes; this constrains the applicability of Cas9 for engineering plant immunity against these viruses.

Recently, novel RNA-targeting CRISPR/Cas effectors, named Cas13, have been shown to specifically bind and cleave RNA rather than DNA substrates. Members of the Class II, type VI CRISPR/Cas13 family have demonstrated a unique ability to efficiently target and degrade single-stranded RNAs when guided by a CRISPR RNA (crRNA) harboring a variable length of targeting (spacer) sequence [21,22,23]. Establishment of this system as an efficient RNA targeting tool has opened exciting opportunities to target and manipulate RNAs [24]. Since their discovery, different Cas13 orthologues have been identified and harnessed for specific RNA manipulations in various systems, including mammalian cells [25], plants [25,26], bacteria [21], and yeast [27].

We have recently demonstrated the utility of a plant codon-optimized Cas13 (pCas13a) orthologue from *Leptotrichia shahii* (LshCas13) to engineer immunity against RNA viruses in *Nicotiana benthamiana* [26]. To illustrate this, Tobacco rattle virus (TRV) was harnessed to systemically express CRISPR RNAs (crRNAs) designed with 28 base pair (bp) sequences complementary to different regions in the Turnip mosaic virus (TuMV) RNA genome in *N. benthamiana* plants stably expressing pCas13a. This targeting resulted in a reduction of up to 50% in viral accumulation, as evident by the lower signal from TuMV-expressed GFP with TuMV-targeting crRNAs compared with non-targeting crRNAs. In addition, we demonstrated the ability of pCas13a to process poly crRNAs in plants to generate functional, mature crRNAs capable of guiding pCas13a to target RNA virus [26]. This Cas13-mediated RNA virus interference thus holds promise as an efficient tool for engineering plant immunity against a broad range of plant RNA viruses.

In this study, we investigated the ability of CRISPR/pCas13a to provide durable and heritable interference against the TuMV-GFP virus in *Arabidopsis thaliana* plants. To implement the system, we engineered *A. thaliana* plants to stably express pCas13a protein and crRNAs targeting different regions in the TuMV-GFP virus genome. Plants with crRNAs targeting the *HC-pro* and *GFP-T2* of the TuMV-GFP RNA genome resulted in efficient virus interference compared with crRNA with no targeting specificity to the TuMV-GFP genome. Our study thus demonstrates the great potential of employing the CRISPR/Cas13 system to engineer stable immunity against RNA viruses in plants. 

## 2. Material and Methods

### 2.1. Cloning of crRNAs under AtU6-26 Promoter

Cas13 crRNAs (GFP-T1, GFP-T2, HC-Pro, and CP) were synthesized and ordered as single-stranded DNA oligos. They were phosphorylated, annealed, and then cloned under the AtU6-26 promoter using BbsI and NcoI enzymes in an intermediate pUC19 vector containing the AtU6-26 promoter. The whole cassette, containing promoter and crRNA was then polymerase chain reaction (PCR) amplified, restricted with SacI enzyme, and ligated into SacI-restricted *pK2GW7:pCas13a* to construct final pK2GW7-p35s::pCas13a-U6-26::crRNA.

### 2.2. Plant Material

*Arabidopsis thaliana* plants expressing pCas13a with crRNAs were generated using the floral dip transformation method. For this purpose, *Agrobacterium tumefaciens* strain GV3101 containing the respective clones were grown in a 5-mL LB culture overnight, which was used to inoculate a large overnight culture of 500 mL. Cells were collected by centrifugation and re-suspended to a final OD of 0.6 in floral dip solution containing 5% sucrose and Silwet at the concentration described by [28]. Mature seeds were harvested and selected on Murashige and Skoog medium containing kanamycin. 

### 2.3. Arabidopsis Infection by TuMV-GFP via Sap Inoculation and Imaging under Ultraviolet (UV) Light

For the sap infiltration method, 3-week-old wild type *Nicotiana benthamiana* plants were infiltrated with Agrobacterium strain GV3010 containing TuMV-GFP infectious clone as shown previously [26]. Systemic leaves were harvested 5–7 dpi and effectively smashed in KH_2_PO_4_ buffer. Next, single leaves of 3-week-old *A. thaliana* transgenic permanent lines overexpressing Cas13 protein and crRNAs were dusted with carborundum of size 200–450 mesh. Leaves were then applied with equal amounts of sap containing TuMV-GFP virus and spread uniformly on the leaf surface. Plants were kept in the growth chamber for 7–10 days then imaged. TuMV-GFP systemic spread was observed in the dark using handheld ultraviolet (UV) light as previously described [26]. Images were taken and systemic leaf samples were collected for molecular analysis.

### 2.4. Immunoblot Analysis 

Immunoblotting was performed as reported previously [26]. Briefly, 100 mg of ground tissue was used for protein extraction. Total proteins were resolved on 10% SDS page. For blotting, rabbit anti-GFP antibody (abcam, Cambridge, UK) (for virus-expressed GFP detection) or rat anti-HA antibody (Roche, Darmstadt, Germany) (for pCas13a detection) at 1:3000 or in 1:1000 dilutions, respectively, were used. All blots were treated with their respective secondary antibodies. ECL detecting reagent from Thermo Scientific was used to detect the signal using a ChemiDoc machine (Bio-Rad, Hercules, CA, USA).

### 2.5. RNA Isolation and Northern Blot Analysis 

RNA extraction and northern blot analysis were performed as previously described [26]. Total RNA was extracted from virus-infected plants using the Direct-zol RNA MiniPrep Plus (Zymo Research, Irvine, CA, USA) according to the manufacturer’s recommendations. For each sample, 10 μg of RNA was separated on a denaturing 1.5% agarose gel, blotted on a Hybond-N+ (GE Healthcare, Chicago, IL, USA) membrane and hybridized with a DIG-labelled probe. For virus expression analysis, a DIG-labelled RNA probe was synthesized using DIG Northern Starter Kit (Roche) and manufacturer’s instructions were followed. For each sample, three independent biological replicates were used. The average knockdown was calculated using Image J software (1.52a, National Institute of Health, Bethesda, Maryland, USA).

## 3. Results

### 3.1. Construction, Over-Expression and Detection of the Clustered Regularly Interspaced Short Palindromic Repeats (CRISPR)/pCas13a Machinery in *A. thaliana* Plants

We previously reported the successful utilization of CRISPR/LshCas13a to target and interfere with the TuMV-GFP RNA virus in *N. benthamiana* plants [26]. We showed the ability to reprogram and direct the Cas13a effector to specifically target the RNA genome of TuMV-GFP virus with virus-targeting crRNAs delivered into *N. benthamiana* plants constitutively expressing pCas13a via our TRV system [29]. Our aforementioned report described the construction and assembly of the LshCas13a into a destination binary vector for in planta expression. 

**Figure 1:**
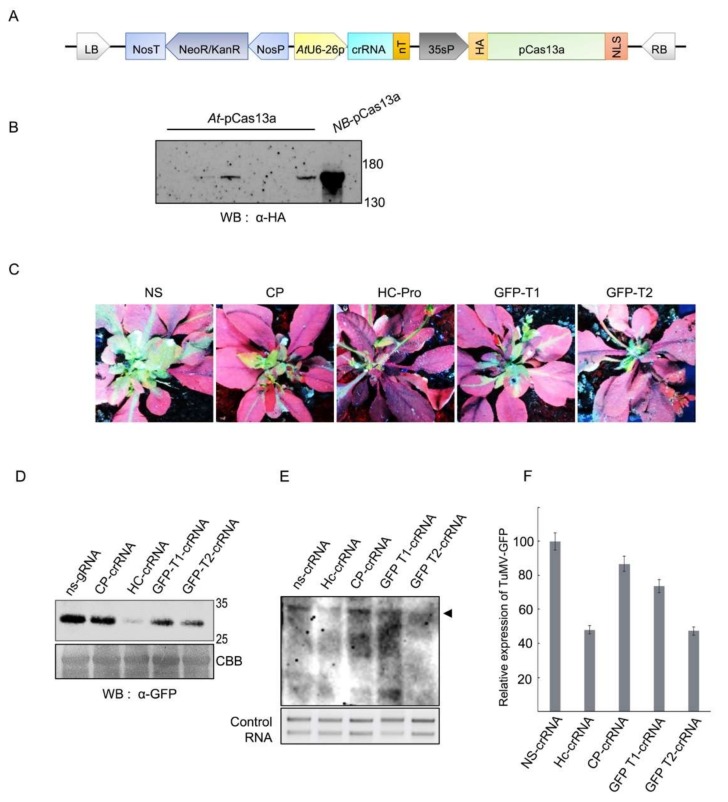
Cas13a interference with TuMV-GFP in *A. thaliana* plants. (**A**) Schematic representation of the engineered system for in planta expression. The T-DNA consists of pCas13a and crRNA driven by the 35s and AtU6-26 promoter, respectively. In addition, it also contains Kan^r^ (kanamycin resistance) driven by the Nos promoter for selection of transgenic plants. (**B**) Confirmation of pCas13a protein expression in T_1_ *A. thaliana* plants. Western blot analysis with anti-HA antibody was used to detect the expression of Cas13a in T_1_ *A. thaliana* lines. NB-pCas13a represents protein extracted from transiently expressed pCas13a in *Nicotiana benthamiana*. (**C**) Interference of TuMV-GFP in pCas13a expressing transgenic *Arabidopsis* lines. Stably transformed *Arabidopsis* lines expressing pCas13 and crRNA were inoculated with TuMV-GFP sap from *N. benthamiana*. Plants were imaged for GFP fluorescence to examine TuMV-GFP systemic spread under UV light in the dark. (**D**) Western blot analysis to confirm TuMV-GFP accumulation in *Arabidopsis* systemic leaves. Protein resolved on 10% sodium dodecyl sulfate polyacrylamide gel electrophoresis (SDS-PAGE) was blotted with GFP antibody to detect virus accumulation. Coomassie brilliant blue (CBB) stained membrane (lower panel) was used as loading control. (**E**) Northern blot to analyze TuMV-GFP virus titer in plants. Northern blot confirms that Hc-crRNA and GFP-T2 crRNAs give better interference with TuMV-GFP followed by GFP-T1 and CP crRNAs. RNA blots from (C) were probed with a DIG-labeled TuMV complementary (250-nt) RNA fragment and detected with anti-DIG antibody. The arrow indicates the accumulation of the TuMV-GFP RNA genome. (**F**) Quantification of TuMV-GFP RNA genome. The graph represents the relative expression of the TuMV-GFP RNA virus as calculated on the bases of three independent biological replicates of northern blot. Error bars represents STDEV.

TuMV harbors a positive, single-stranded RNA genome of about 10,000 nucleotides encoding around 10 different functional proteins [30]. In addition, a GFP-encoding sequence was introduced into the TuMV genome, making the virus’s replication and movement easy to assay and monitor. In this study, we used the *pK2GW7-p35s::pCas13a* over-expression clone as a destination vector to insert cassettes containing single crRNAs complementary to sequences within the TuMV-GFP RNA genome under the *AtU6-26* polymerase III promoter, generating binary vectors expressing pCas13a and crRNAs (*pK2GW7-p35s::pCas13a-U6-26::crRNA*) (Figure 1A). Four different target regions within the TuMV-GFP RNA genome were selected: two targets within the *GFP* sequence (GFP-T1 and GFP-T2), the helper component proteinase silencing suppressor (*HC-Pro),* and the coat protein (CP) (Table 1). Subsequently, *Agrobacterium* GV3101 cultures harboring the *pK2GW7-p35s::pCas13a-U6-26::crRNA* binary clones were used to transform *Arabidopsis thaliana* plants via the floral dip method. pCas13a expression was confirmed in T_1_ transgenic plants with Western blotting, and a protein of the expected size was detected (Figure 1B).

### 3.2. CRISPR/pCas13a-Mediated TuMV-GFP Virus Interference in *A. thaliana* Plants 

To explore whether CRISPR/pCas13a could provide heritable interference against TuMV-GFP infection in transgenic *A. thaliana* plants, we followed the plants from the T_0_ generation (transformed with CRISPR/Cas13 constructs), to the T_2_ generation for the presence of the T-DNA (seeds were selected on kanamycin-containing media, which indicates T-DNA integration in the plant genome). T_2_ seeds of T_1_ plants positive for the pCas13a expression were kanamycin selected and chosen for viral interference experiments. Plants expressing pCas13a with the virus-targeting crRNAs were infected with TuMV-GFP virus by the sap inoculation method. In addition, *A. thaliana* plants expressing pCas13a and a non-specific crRNA (ns-crRNA) with no sequence complementarity to the TuMV-GFP genome was used as a control. The interference activity of CRISPR/pCas13a against the virus infection can be assayed by visualizing the TuMV-expressed GFP in the systemic leaves, which reflects viral replication and movement. At 7–10 days post TuMV-GFP infection (dpi), reduction in GFP intensity was clearly observed in plants with crRNAs targeting *HC-pro* and *GFP* (GFP-T2) regions compared with the control, indicating that TuMV-GFP infection was significantly attenuated by CRISPR/pCas13a system (Figure 1C). Other crRNAs including GFP-T1 and CP targeting crRNAs were less efficient, suggesting that different factors, including secondary structures within the virus genome, or proteins bound to the virus RNA genome may influence the activity of CRISPR/pCas13a (Figure 1C). 

Western blotting using the anti-GFP monoclonal antibody was performed to validate the observed reduction in the expression level of the virus-expressed GFP in systemic leaves. The results of Western blotting showed a significant decrease in the GFP level in plants with crRNAs targeting the TuMV-GFP virus compared with control, confirming the results obtained from the analysis of the GFP signals (Figure 1D). In addition, quantification of the TuMV-GFP virus titer with northern blotting showed a considerable reduction in the accumulation of viral RNA genomes, specifically with HC-pro and GFP-T2 crRNAs, consistent with the reduction observed in the level of GFP expression in plants with virus-targeting crRNAs compared with the control (Figure 1E,F). Taken together, these results demonstrate that pCas13a guided by virus-targeting crRNAs can effectively target and interfere with the TuMV-GFP virus replication, and thus suppress its infection and systemic movement in *Arabidopsis* plants.

## 4. Discussion 

Plant viruses threaten agriculture by significantly affecting plant growth and yield, and thus compromising food security around the world. Each year, pathogenic plant viruses are estimated to cause around a 10–15% reduction in crops yield worldwide [31]. Engineering genetic resistance in plants against viruses is one of the most sustainable approaches to control virus infections [4]. Over the past few years, the bacterial adaptive immunity CRISPR/Cas systems have been adopted as powerful genome engineering tool in various systems, including plants [32]. 

**Figure 2:**
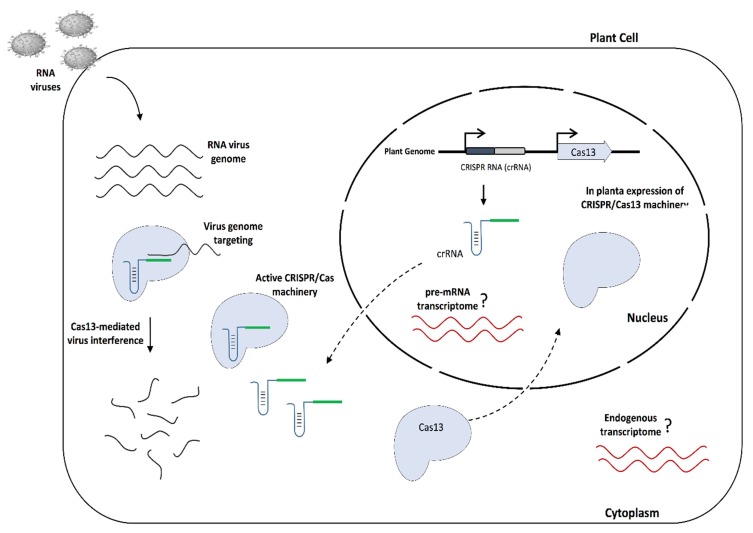
A schematic representation of CRISPR/Cas13-mediated RNA virus interference in plants. The diagram illustrates the mechanism of molecular immunity against RNA viruses in *A. thaliana* plants expressing CRISPR/Cas13a. When plants are infected with virus, Cas13 guided by virus-targeting crRNA recognizes and degrades the RNA virus genome, providing immunity against the virus.

We have recently shown the establishment of the CRISPR/LshCas13a system to provide molecular immunity against the potyvirus Turnip mosaic virus (TuMV) in *N. benthamiana* plants [26]. The TuMV-GFP virus was targeted and knocked down with different crRNAs that were delivered into *N. benthamiana* plants stably expressing the pCas13a protein via the TRV system. However, although efficient virus interference was achieved, the transient TRV-mediated delivery of crRNAs did not allow us to test heritable resistance against the virus in the next generations. Here, we aimed to expand the utility of CRISRP/pCas13a to provide durable and heritable RNA virus interference in plants by engineering *A. thaliana* plants stably expressing the pCas13a protein and crRNAs targeting different regions of the TuMV-GFP virus genome (Figure 2). 

We initially tested the CRISPR/Cas13-mediated virus interference in T_1_ plants and observed the virus interference, most notably for HC and GFP-T2-expressing lines. Since this is a gain of function, this engineered activity should be inherited in the next generations. To test this idea, we used T_2_ plants selected on kanamycin to confirm the ability of CRISPR/Cas13 to provide virus interference in the next generation. Similar to results observed in T_1_ plants, we observed effective interference against the TuMV-GFP virus, particularly with plants expressing Cas13 and crRNAs targeting the HC and GFP regions in the TuMV-GFP RNA genome. Although we assayed the RNA virus interference ability of CRISPR/pCas13a up to the T_2_ generation, the stable integration and expression of both Cas13a and its cognate crRNAs in the same cell in transgenic *A. thaliana* plants would confer the heritability of the CRISPR/Cas13a-mediated immunity in the next generations. 

Several studies have shown that different crRNAs result in different interference efficiencies, largely due to the accessibility of the target RNA by the Cas13:crRNA complex [21,25]. Here, crRNAs targeting GFP-T1 and CP were less effective in comparison to GFP-T2 and HC-pro. Interestingly, the variability in the efficiencies of different virus-targeting crRNAs in this study were consistent with the results observed in our previous report, where GFP-T2 and HC-pro targeting crRNAs were more efficient than other crRNAs, including GFP-T1 and CP targeting crRNAs [26]. These results validate the utility of harnessing the TRV system to test and compare different crRNAs simultaneously and in parallel before the generation of transgenic plants expressing Cas proteins and their cognate crRNA/gRNAs. 

Off-target activity of CRISPR/Cas systems has been one of the major concerns associated with the utilization of this technology. In addition, DNA delivery of the Cas protein and its gRNAs into plants might increase the likelihood of off-target activity [33], which could be true in the case of CRISPR/Cas13. In line with this notion, we did not observe any phenotypic or growth defects in these lines prior to virus infection under normal conditions, indicating little or no significant off-target activity.

We have previously shown the ability of pCas13a to process long poly-crRNAs to generate mature and functional crRNAs capable of guiding Cas13a to its target RNA in plants. Engineering plants that can stably express Cas13a and poly-crRNAs targeting different RNA viruses would provide plants with stable immunity to different viruses, which is of great importance especially for plants exposed to mixed infections of epidemic viruses [34]. Lastly, recent findings have shown the characterization of novel and more robust RNA-targeting Cas13 variants. It would be interesting to test and compare different Cas13 variants to determine and establish more efficient RNA virus immunity in plants.

Our study demonstrates the exciting potential of CRISPR/Cas13 for use as an antiviral strategy to obstruct the aggressive and crop-threatening RNA viruses, and encourage the search for and the development of more robust and effective RNA targeting CRISPR systems.

## Figures and Tables

**Table 1:** Oligos used in this study.

Oligo Name	Sequence (5′-3′)	Purpose
GFP-T1-U6-Top strand	gattgccaccccaatatcgaaggggactaaaacaacaggtagttttccagtagtgcaaatatttttttttc	TuMV-GFP-T1 crRNA
GFP-T1-U6-Bottom strand	catggaaaaaaaaatatttgcactactggaaaactacctgttgttttagtccccttcgatattggggtggc	
GFP-T2-U6-Top strand	gattgccaccccaatatcgaaggggactaaaacccgtcctccttgaaatcgattcccttaatttttttttc	TuMV-GFP-T2 crRNA
GFP-T2-U6- Bottom strand	catggaaaaaaaaattaagggaatcgatttcaaggaggacgggttttagtccccttcgatattggggtggc	
HC-Pro-U6-Top strand	gattgccaccccaatatcgaaggggactaaaacccgcttgcttgtccttgggatagctcactttttttttc	TuMV-HC-pro crRNA
HC-Pro-U6- Bottom strand	catggaaaaaaaaagtgagctatcccaaggacaagcaagcgggttttagtccccttcgatattggggtggc	
CP-U6-Top strand	gattgccaccccaatatcgaaggggactaaaacacactgaaagttccagaggttccagcgttttttttttc	TuMV-CP crRNA
CP-U6-Bottom strand	catggaaaaaaaaaacgctggaacctctggaactttcagtgtgttttagtccccttcgatattggggtggc	
AtU6-F	atgagctcaagcttcgacttgccttccg	For PCR amplification of AtU6 promoter-crRNA cassette out of the intermediate pUC19 vector
AtU6-R	aggtaccacgcgtcactagtataaccatgg	
